# The pathophysiology of cancer‐mediated cardiac cachexia and novel treatment strategies: A narrative review

**DOI:** 10.1002/cam4.6388

**Published:** 2023-09-01

**Authors:** Louisa Tichy, Traci L. Parry

**Affiliations:** ^1^ Department of Kinesiology University of North Carolina Greensboro Greensboro North Carolina USA

**Keywords:** cachexia, cancer, cardiac cachexia, heart, muscle wasting

## Abstract

**Significance:**

Two of the leading causes of death worldwide are cancer and cardiovascular diseases. Most cancer patients suffer from a metabolic wasting syndrome known as cancer‐induced cardiac cachexia, resulting in death in up to 30% of cancer patients. Main symptoms of this disease are severe cardiac muscle wasting, cardiac remodeling, and cardiac dysfunction. Metabolic alterations, increased inflammation, and imbalance of protein homeostasis contribute to the progression of this multifactorial syndrome, ultimately resulting in heart failure and death. Cancer‐induced cardiac cachexia is associated with decreased quality of life, increased fatiguability, and decreased tolerance to therapeutic interventions.

**Recent advances:**

While molecular mechanisms of this disease are not fully understood, researchers have identified different stages of progression of this disease, as well as potential biomarkers to detect and monitor the development. Preclinical and clinical studies have shown positive results when implementing certain pharmacological and non‐pharmacological therapy interventions.

**Critical issues:**

There are still no clear diagnostic criteria for cancer‐mediated cardiac cachexia and the condition remains untreated, leaving cancer patients with irreversible effects of this syndrome. While traditional cardiovascular therapy interventions, such as beta‐blockers, have shown some positive results in preclinical and clinical research studies, recent preclinical studies have shown more successful results with certain non‐traditional treatment options that have not been further evaluated yet. There is still no clinical standard of care or approved FDA drug to aid in the prevention or treatment of cancer‐induced cardiac cachexia. This review aims to revisit the still not fully understood pathophysiological mechanisms of cancer‐induced cardiac cachexia and explore recent studies using novel treatment strategies.

**Future directions:**

While research has progressed, further investigations might provide novel diagnostic techniques, potential biomarkers to monitor the progression of the disease, as well as viable pharmacological and non‐pharmacological treatment options to increase quality of life and reduce cancer‐induced cardiac cachexia‐related mortality.

## 1 INTRODUCTION

Cancer and cardiovascular diseases are two of the leading causes of death worldwide and the burden of these diseases is growing rapidly.^1^ Up to 80% of cancer patients suffer from a multiorgan, metabolic wasting syndrome known as cancer cachexia, which results in the death of up to one third of these cancer patients.^2^ Besides the main symptom of skeletal muscle and adipose tissue wasting, most cachectic cancer patients suffer from cardiac atrophy, remodeling and dysfunction, known as cancer‐induced cardiac cachexia, which ultimately leads to heart failure and death.^3^ Metabolic alterations, increased systemic inflammation, as well as dysfunctional protein homeostasis are causing these weight loss, muscle loss, and dysfunctional symptoms of cancer‐mediated cachexia, which cannot be fully reversed by nutritional interventions.^4^ While research is growing in the field, the molecular mechanisms behind this disease are not well understood and there are no clear diagnostic criteria to identify the development and progression of cancer cachexia and cancer‐mediated cardiovascular impairments. Research has shown that certain nutritional, exercise, and pharmacological interventions may slow the progression of this disease. However, cancer‐mediated cardiac cachexia remains an untreated condition with irreversible effects and significantly reduces prognosis of survival and overall quality of life of cachectic cancer patients.^3^, ^5^ While other reviews have summarized the identified traditional treatment interventions, including beta‐blockers, and other heart failure medications,^2^, ^6^ there is still no standard of care for cancer‐mediated cardiac cachexia. Many questions remain regarding the most effective timing and combination of individualized pharmacological and non‐pharmacological treatment plans for cancer patients to prevent or reverse the detrimental effects of this disease. The purpose of this narrative review was to revisit the currently known underlying molecular mechanisms, signaling pathways, and potential biomarkers, as well as adding new knowledge and recent discoveries of novel, non‐traditional preclinical and clinical treatment approaches that show the potential to aid in the treatment of cancer‐induced cardiac cachexia.

## 2 CANCER‐INDUCED CARDIAC CACHEXIA

Worldwide, cardiac cachexia affects approximately 26 million people. Cardiac cachexia in general is associated with atrophy of cardiomyocytes, changes in myocardial structure, myocardial remodeling, dysfunctional cardiac metabolism, and overall decreased cardiac function, which ultimately results in heart failure and death.^7^, ^8^, ^9^ Many disease states can induce cardiac cachexia, such as pulmonary hypertension, chronic obstructive pulmonary disorder or heart failure,^5^, ^10^ but one of the most understudied subtypes remains cancer‐induced cardiac cachexia. Up to 40% of the cancer patients and most cachectic animals experience cardiac muscle wasting and dysfunction in addition to other symptoms of cancer cachexia, such as skeletal muscle wasting.^9^, ^11^ Cancer‐induced cardiac abnormalities increase the risk of mortality and are the primary cause of death in up to 30% of the cancer patients, most seen in colorectal, endometrial, breast, melanoma, prostate, and urinary bladder cancers.^12^, ^13^

Most research suggests that cardiac dysfunction and muscle wasting is induced by dysbalanced metabolic homeostasis leading to a catabolic shift during cancer‐induced cachexia.^3^, ^14^ Besides the typical symptoms and clinical presentations of cachexia, including weight loss, decreased skeletal muscle mass and strength, as well as decreased appetite or anorexia, cachectic cancer patients suffering from cardiac abnormalities and atrophy present with symptoms of decreased cardiac function or heart failure.^7^, ^12^, ^14^ While the pathophysiology of heart failure in cancer‐induced cardiac cachexia is not fully understood, certain risk factors of developing cardiac cachexia during cancer are comparable to general cancer cachexia risk factors, including age, sex, and lifestyle factors. Additionally, cardiovascular‐specific factors that increase the risk of developing cardiac muscle wasting during advanced cancer stages include hypertension, smoking, stress, and dyslipidemia prior to cancer diagnosis.^5^, ^15^ Despite known risk factors and hallmark signs, there are still no clear diagnostic criteria or treatment options for cancer‐induced cardiac cachexia. However, multimodal studies, implementing combinations of pharmacological and non‐pharmacological interventions, have shown promising results in reducing or slowing the development of this disease.^5^, ^16^, ^17^

### 2.1 Pathophysiology of cancer‐induced cardiac cachexia

At the center of the pathophysiology of cancer cachexia is metabolic dysfunction characterized by an imbalance of abnormally increased catabolism and decreased anabolism.^18^ Multiple factors can induce metabolic dysfunction or malnutrition during cancer cachexia, including adverse effects of chemotherapy, obstructive effects of local tumors, tumor cells competing for available nutrients, and energy fuels leading to altered energy balance in healthy tissues, as well as an additional overall increase in energy demand due to the uncontrollable growth patterns of tumor cells.^11^, ^15^, ^19^ This imbalance and metabolic shift results in the hallmark sign of cancer cachexia, cancer‐induced skeletal muscle wasting—with or without the loss of fat mass.^20^

The pathophysiology of cancer‐induced cardiac cachexia is very complex and not fully understood. Different preclinical studies using C26 tumor models in mice have shown metabolic shifts in cardiac tissue due to the whole‐body metabolic imbalance. Tian et al. identified altered gene expression in tumor‐bearing mice indicative of cardiac remodeling, including increased BNP, decreased PPARα and a shift in MHC isoforms from an “adult” to an “embryonic” phenotype.^21^, ^22^ This isoform switch is associated with increased glucose utilization, decreased fatty acid oxidation, systolic dysfunction, mitochondrial dysfunction, and overall maladaptation and worsening of heart function.^6^, ^11^, ^23^ In a proteomic study of a C26 tumor mouse model, MHC isoform switch and a decreased total expression of myosin heavy chain proteins was associated with destabilized sarcomeres of cardiac myocytes. These findings were indicative of increased sarcomeric protein release for degradation (e.g., desmin) to overcome the metabolic stress on the cardiovascular system due to tumor burden.^24^

**FIGURE 1:**
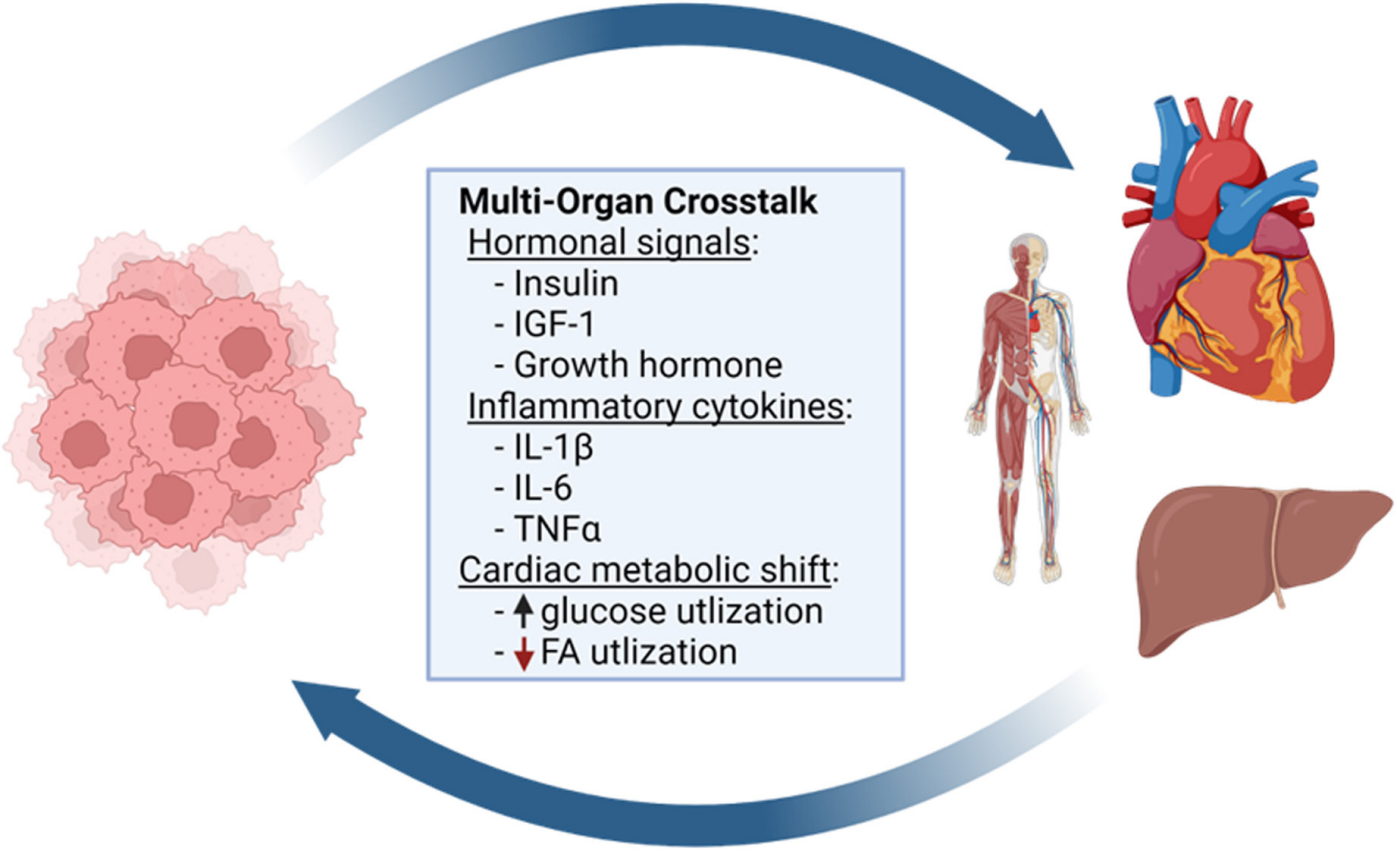
Multiorgan crosstalk in cancer‐mediated cardiac cachexia. During cardiac cachexia, it is suspected that both the heart and the tumor act as secondary endocrine glands to release and regulate the release of hormones and inflammatory cytokines. This leads to tissues like the heart altering their metabolism in response to this multiorgan signaling cascade. The heart notoriously shifts away energetically favorable fatty acid metabolism and toward energetically unfavorable glucose metabolism.

Preclinical studies of cardiac cachexia induced by different cancer models have also suggested a crosstalk between tumor and cardiac tissue found via circulating molecules (Figure 1). Both tumor and cardiac cells can be affected by or release these factors. Tumor cells as well as cardiac cells can act as endocrine organs by releasing or affecting the release of hormonal signals such as insulin, IGF‐1, growth hormone, as well as inflammatory cytokines. Several of these factors can lead to metabolic dysfunction and increased systemic inflammation, as well as affect tumor growth.^3^, ^7^, ^11^, ^15^ Cancer‐mediated depletion of insulin, for example, causes dysfunctional glucose metabolism, leading to decreased glucose uptake in the cardiac tissue resulting in cardiac muscle wasting. Insulin deficiency has been reported in many cachectic cancer patients and cachectic rodents in advanced stages of tumor burden.^25^, ^26^ Thackeray et al.^26^ discovered that B16F10 and C26 tumor cells in mice induced production of insulin‐degrading enzymes, lowering of pancreatic insulin production, and ultimately a shift of glucose away from the cardiomyocytes by increasing glucose consumption within the tumor cells. These findings indicate systemic tumor‐mediated metabolic shifts leading to cardiac atrophy, MHC shift, and decreased cardiac function. However, insulin supplementation was able to attenuate tumor‐mediated effects on the cardiac tissue.^26^ Other cancer cell‐derived cachexokines, inflammatory cytokines, and metabolic messengers, such as ataxin‐10, IL‐6, and D2‐HG, are also involved in metabolic alterations, dysfunctional glucose uptake, promotion of tumor growth, and increased cardiac muscle wasting.^27^, ^28^, ^29^

Conversely, a study by Dostal et al.^30^ looking at cardiac function in a C26 adenocarcinoma‐induced cachexia mouse model, was one of the first to show that tumor burden rather than cancer cachexia‐mediated skeletal muscle and fat wasting played a significant role in the progression of cardiac dysfunction. Abnormal functional changes in the hearts of tumor‐bearing mice were associated with reductions in heat shock proteins Hsp70 and BCL‐2‐associated athanogene 3 (BAG3), both known for their roles in regulation of protein quality control and maintenance of structural integrity in the heart and vasculature.^30^, ^31^

The combination of increased systemic inflammation and metabolic dysfunction during cancer‐induced cardiac cachexia can lead to an increase in energy expenditure of about 15%, where energy demand significantly exceeds energetic capacity resulting in cardiac atrophy, remodeling, and dysfunction.^19^, ^32^, ^33^ Overall, cardiac muscle wasting due to cancer‐induced cardiac cachexia is associated with systemic and cardiac metabolic dysfunction, inflammation and ROS production, increased energy expenditure and demand.^26^, ^28^, ^29^, ^33^, ^34^

### 2.2 Cardiac dysfunction during cancer‐induced cardiac cachexia

**FIGURE 2:**
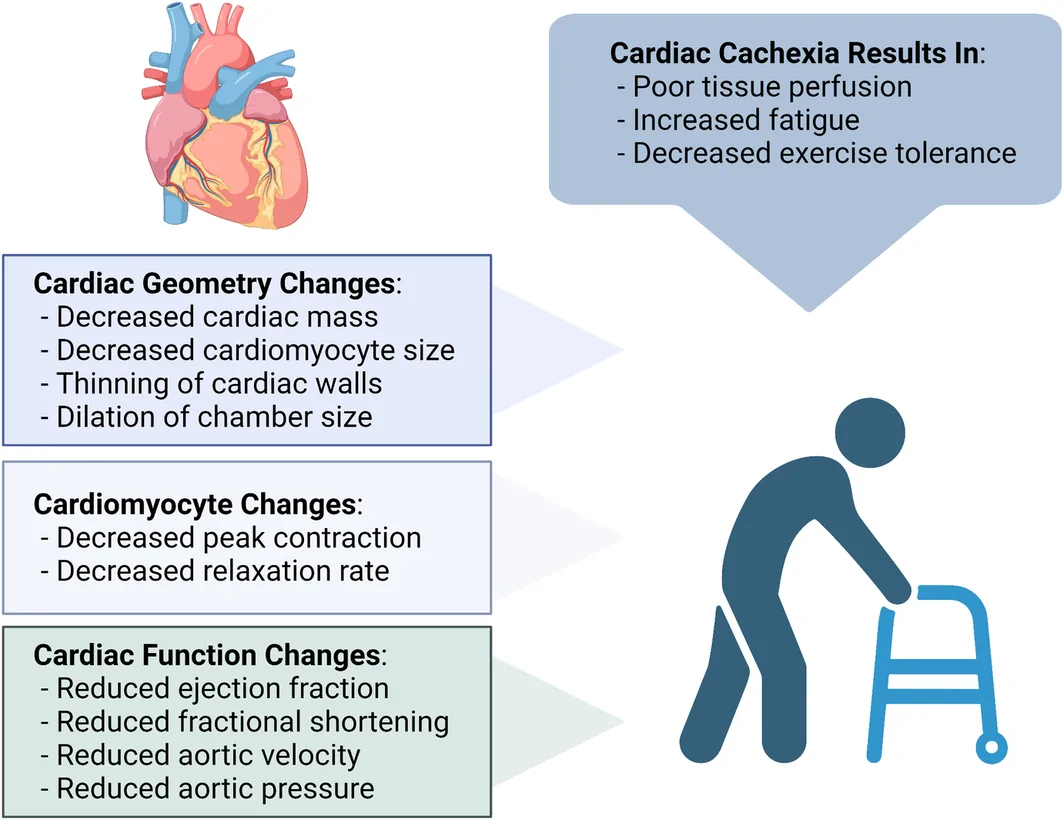
Cardiac clinical presentation of cancer‐mediated cardiac cachexia. Cancer‐mediated changes in cardiac structure and in cardiomyocyte contraction and relaxation result in significant declines in cardiac function. Together, these changes in cardiac structure and function lead to poor tissue perfusion and increased fatigue in cancer survivors with cardiac cachexia.

The metabolic imbalance and shift toward catabolism, altered energy balance, and increase in inflammation due to tumor burden result in cardiac dysfunction in cancer patients and cachectic animals in preclinical studies. The most common cardiac impairments found in clinical and preclinical studies are reduction in cardiac mass, impaired cardiac function, specifically left ventricular function, and cardiac remodeling (Figure 2).^35^

Preclinical studies on rodents with different tumor models have repeatedly demonstrated cancer‐induced cardiac muscle wasting, cardiac dysfunction, and cardiac remodeling. Multiple studies of C26 adenocarcinoma injections in CD2F1 or BALB/C mice revealed systolic dysfunction, depressed cardiomyocyte function during contraction and relaxation phases, and cardiac remodeling via in vivo echocardiography.^36^, ^37^, ^38^ Reduction in cardiac function characterized by cardiac atrophy, impaired left ventricular ejection fraction (LVEF), and fractional shortening, as well as cardiac necrosis, inflammation, and fibrosis has been associated with a systemic effect of tumor burden on the heart.^36^, ^37^ Another study within the C26 mouse tumor model identified significantly reduced cardiac function at the cellular level of cachectic hearts in an isolated perfused heart model. Ex vivo perfused hearts exhibited contraction and relaxation deficits, decreased peak contraction and relaxation rates, as well as systolic and diastolic dysfunction characterized by decreased left ventricular (LV) developed pressure and prolonged time to 50% pressure fall.^39^ Isolated cardiac myocyte width and myofibril number was significantly decreased, explaining the decreased cardiac mass in cachectic mice compared to non‐cachectic controls.^39^, ^40^ Coinciding with these findings, decreased heart mass has been associated with dysfunctional LV pressure changes, abnormal fractional shortening, decreased cardiac output, and persistent arrhythmias in LLC tumor models in C57BL6/C mice and fisher rats inoculated with MatBIII cancer cells.^8^, ^41^, ^42^ One study on CD2F1 mice injected with C26 adenocarcinoma cells identified sex‐based differences in cardiac dysfunction. Male cachectic animals experienced a 16% reduction in aortic velocity and 30% reduction in aortic pressure, whereas female cachectic animals did not show any alterations in cardiac function.^43^

Similar findings have been seen in clinical studies of cardiomyopathies in cachectic cancer patients. The most common clinical presentations associated with cancer‐induced cardiac cachexia are comparable to chronic heart failure symptoms, including fatigue, dyspnea, anemia, and decreased exercise tolerance.^44^ Clinical diagnostic assessment and monitoring studies for cancer‐induced cardiac cachexia specifically have not yet been well defined or optimized. Few retrospective or longitudinal studies have compared cardiac function of cachectic cancer patients with chronic heart failure patients and healthy controls.^12^, ^20^, ^45^ Colorectal, lung, and gastrointestinal cancer patients suffering from cachexia showed significantly smaller heart weights and impaired LVEF compared to non‐cachectic or healthy controls.^12^, ^20^ Cramer et al.^12^ found that cachectic cancer patients exhibited increased cardiac posterior wall thickness and decreased LVEF. However, LVEF was not as impaired as ejection fraction of chronic heart failure (CHF) patients.^12^ A recent study by Lena et al.^45^ compared cardiac mass and function of 300 patients with active, advanced cancer but without cardiovascular disease or infection to healthy controls and CHF patients. Independent of cardiotoxic anticancer treatment, cancer patients showed significantly lower LV masses compared to CHF patients and healthy controls. Specifically, LV mass was lowest in cachectic cancer patients. Follow‐up echocardiography identified further declines in LV mass and stroke volume, as well as increased resting heart rates in surviving cancer patients.^45^ Overall, low LV mass in cachectic cancer patients and cachectic animals has been associated with poor cardiac function and increased all‐cause mortality. This growing body of literature of clinical evidence of cardiac wasting‐associated cardiomyopathy in cancer coincides with many preclinical studies that have repeatedly demonstrated cancer‐induced cardiac muscle wasting.

Overall, cancer‐mediated cardiac cachexia is associated with cardiac atrophy, cardiac remodeling, and decreased cardiac function leading to decreased oxygen supply throughout the body, increased fatigue and hallmark symptoms of heart failure.^2^, ^46^ Collectively, clear diagnostic criteria, measurements, and clinical values are lacking. Diagnostic criteria will likely need to be a combination of measurements, for example functional measures (e.g., ejection fraction) and structural measures (e.g., wall thickness and chamber diameter), in order to accurately identify at risk patients and progression of cancer‐mediated cardiac cachexia.

### 2.3 Molecular mechanisms of cancer‐mediated cardiac cachexia

Due to the complexity of cancer‐induced cardiac cachexia, the molecular mechanisms resulting in the pathophysiological characteristics of this disease are not well understood. However, anorexia, altered energy balance, and an abnormal metabolism associated with increased protein degradation and decreased protein synthesis, as well as inflammation induced by the tumor and immune system seem to be key players in the development and progression of this disease.

Other reviews have summarized general pathways involved in cardiac cachexia, non‐specific to cancer‐induced cardiac cachexia. These include altered cardiac metabolism via inflammation induced by an increase in pro‐inflammatory cytokines, such as TNFα, IL‐1β, and IL‐6, and a decrease in anti‐inflammatory markers, such as IL‐10. The elevated release of pro‐inflammatory cytokines promotes the process of cachexia by regulating the major pathways involved in muscle wasting.^2^, ^3^, ^6^, ^11^, ^15^, ^17^ IL‐6 and TNFα are two of the primary mediators of general cardiac cachexia by suppressing protein synthesis and promoting increased energy expenditure via upregulation of inflammatory processes.^47^, ^48^

While the pathways specific to cancer‐induced cardiac cachexia are still understudied and not well understood, few preclinical tumor models have identified the inflammatory and metabolic pathways specific to cardiac cachexia during cancer. Similar to general cardiac cachexia, the pro‐inflammatory cytokines IL‐6 and TNFα are upregulated in the heart in different cancer cachexia‐inducing rodent models.^28^, ^31^, ^49^ Factors from the TGF‐β family, such as activin A, myostatin, or GDF15, have also been shown to induce cancer‐mediated cardiac cachexia in tumor models.^10^, ^50^, ^51^ These markers are involved in the induction of protein degradation pathways by binding to their respective cell‐surface receptors and activating NF‐κβ, STAT3 and SMAD2/3 pathways. This leads to an increased expression of two E3 ubiquitin ligases, MuRF1 and Atrogin‐1, which accelerates the protein degradation process through the ubiquitin–proteasome system (UPS) and autophagy, inducing cardiac muscle wasting and remodeling.^6^, ^52^, ^53^ Specifically, GDF‐15, one of the key players in skeletal and cardiac muscle wasting, is a promising biomarker for disease outcome and potential target for treating cancer‐induced cardiac cachexia. Many recent preclinical and clinical studies have identified GDF‐15 as a stress‐hormone released during cancer. Abnormally increased GDF‐15 levels in the circulation of cachectic cancer patients and rodents have been negatively associated with disease outcome and death.^10^, ^50^, ^54^, ^55^, ^56^

**FIGURE 3:**
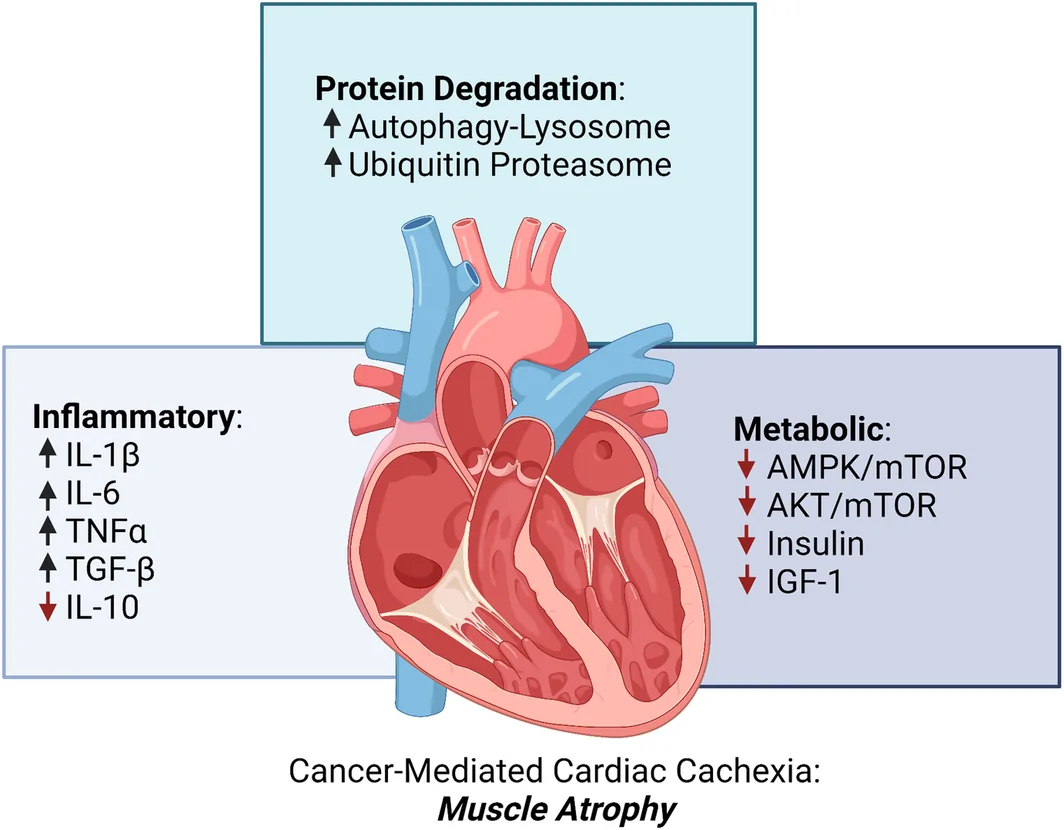
Mechanisms involved in cancer‐mediated cardiac cachexia. While the molecular mechanisms underlying cancer‐mediated cardiac cachexia are not well understood, the upregulation of pro‐inflammatory markers and downregulation of anti‐inflammatory markers appear to lead to the enhanced activation of degradation processes, such as autophagy. The concomitant inhibition of protein synthesis via multiple energy‐ and hormone‐dependent pathways resulting in deactivation of mTOR signaling.

While the three major pathways for protein catabolism during cachexia are the UPS pathway, calcium‐activated system, and autophagy, the autophagy–lysosomal pathway plays the most important role in cancer‐induced cardiac cachexia compared to skeletal muscle cachexia. It has been shown that cachectic animals as well as cachectic cancer patients experienced an increased expression of autophagic markers, such as Beclin‐1, p62, or LC3B‐II.^3^, ^43^, ^44^, ^46^, ^53^ Besides the upregulation of these protein degradation pathways, protein synthesis pathways are downregulated or suppressed during cardiac cachexia, resulting in the disruption of protein homeostasis, cardiac atrophy, and ultimately heart failure. Two of the main protein synthesis pathways that are downregulated or inhibited in this process are the energy‐dependent AMPK/mTOR pathway as well as the insulin‐dependent and IGF‐1‐mediated AKT/mTOR pathway^57^, ^58^ (Figure 3). Constant competition for energy availability, uptake of nutrients, and utilization of glucose by tumor cells, as well as increased systemic inflammation leads to downregulated circulation of insulin and IGF‐1 and decreased energy availability resulting in the downregulation or inhibition of AKT and the deactivation of mTOR. Apc^Min/+^ mice developing colorectal cancer‐induced cardiac cachexia with diminished heart mass compared to controls exhibited decreased myofibrillar protein synthesis. This was accompanied by decreased mTOR phosphorylation and increased Beclin‐1 protein expression in cardiac tissue.^57^ Similarly, tumor‐bearing rodents in a rat hepatoma cancer‐induced cardiac cachexia model exhibited cardiac dysfunction associated with decreased LV protein expression of IGF‐1, insulin, and decreased phosphorylation of AKT and mTOR.^58^ Together, these findings suggest that cancer‐induced cardiac cachexia is associated with the suppression of anabolic signaling and upregulation of catabolic pathways resulting in cardiac muscle wasting and dysfunction.

While the molecular mechanisms behind cancer‐mediated cardiac cachexia are not fully understood, it has been shown that the upregulation of pro‐inflammatory markers and downregulation of anti‐inflammatory markers can lead to the enhanced activation of degradation processes, such as autophagy. Additionally, there is preclinical evidence of inhibition of protein synthesis through multiple energy‐ and hormone‐dependent pathways, including the deactivation of mTOR signaling. Together, these pathways coincide with clinical findings of decreased heart mass, LVEF, and overall decreased cardiac function. While there are no clear diagnostic criteria or biomarkers specific to cancer‐induced cardiac cachexia, most potential biomarkers in the circulation associated with cancer cachexia in general can be promising in the detection of cardiomyopathies, including TNFa, IL‐6, IL‐1b, and IL‐10.^28^, ^49^ Promising cardiac‐specific biomarkers in cancer‐induced cardiac cachexia models and cachectic cancer patients include factors from the TGF‐β family, such as GDF‐15, autophagy markers, such as Beclin‐1, and markers of protein synthesis, such as AKT and mTOR. In cachectic lung cancer patients, high circulating GDF‐15 levels have been associated with weight loss and decreased prognosis of survival.^54^, ^55^, ^59^, ^60^ Although there is emerging evidence of cardiac‐specific biomarkers for cancer‐induced cardiac cachexia, clinical validation studies across different cancer types and cancer stages are needed.

## 3 TREATMENT FOR CANCER‐INDUCED CARDIAC CACHEXIA

**FIGURE 4:**
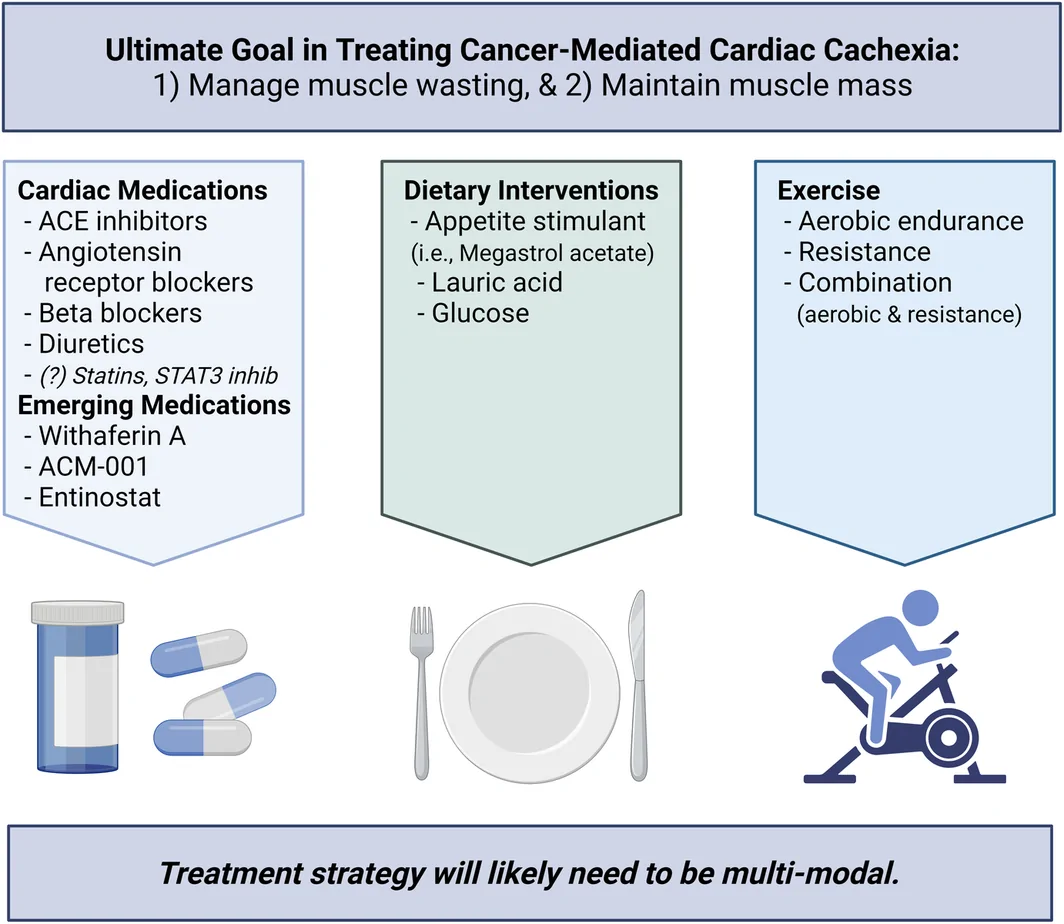
Treating cancer‐mediated cardiac cachexia must be multimodal. Currently, there are no effective and clinically approved treatment options or FDA‐approved drugs available for the treatment and prevention of cardiac cachexia. Due to its multifactorial and complex nature, it has been suggested that one single therapeutic approach may not be successful, and a combination of pharmacological, dietary, and exercise therapies may be the most beneficial treatment strategy. Regardless of intervention, the goal of treating cancer‐mediated cardiac cachexia should focus on managing muscle wasting and maintaining muscle mass.

There are no current effective and clinically approved treatment options or FDA‐approved drugs available for the treatment and prevention of cancer‐induced cardiac cachexia. Due to its multifactorial and complex nature, it has been suggested that one single therapeutic approach may not be successful and a combination of pharmacological and non‐pharmacological therapies may be beneficial (Figure 4).^3^, ^5^

### 3.1 Standard cardiovascular pharmacological treatment strategies

The use of standard pharmacological therapeutic strategies also used for cardiovascular disease and heart failure patients, such as ACE inhibitors, angiotensin receptor blockers, beta blockers, or diuretics, have shown mixed results in patients suffering from cancer‐induced cardiac cachexia as previously reviewed by others.^3^, ^5^, ^6^, ^17^ In heart failure patients, treatment with ACE inhibitors has led to decreased cardiac workload, the prevention of cardiac dysfunction, and the delay in progression of cardiac cachexia by improving endothelial function, regulating IGF‐1 circulating levels, and decreasing expression of inflammatory markers IL‐6 and TNFα, but has not been specifically tested in cancer patients suffering from cardiac cachexia.^3^, ^5^ Angiotensin receptor blockers, such as Losartan, have shown effective results in in vivo and in vitro preclinical cancer cachexia studies by attenuating muscle loss, reducing inflammation via modulation of IL‐6 expression, as well as improving cardiac systolic function.^61^ Treatment with other heart failure medication, such as aldosterone inhibitors or beta‐blockers in rat tumor models, attenuated cardiac cachexia by preserving LV mass, improving LVEF and fractional shortening, as well as downregulating UPS and autophagic pathways.^58^

Other treatments with mixed results include statins and STAT3 inhibitors. Statins as a treatment option for cancer‐induced cardiac cachexia remains controversial. While some studies report positive effects in AH‐130 tumor‐bearing rats, including reduced cardiac weight loss, improved LVEF and SV, other studies of the same tumor model reported negative effects of same statin treatment with exacerbated cardiac weight reductions in treated tumor‐bearing rats.^62^, ^63^ Other preclinical pharmacological treatment approaches in C26 murine tumor models have targeted STAT3 inhibition and reactive oxygen species attenuation. This resulted in decreased cardiac muscle wasting, restored left ventricular function and restored mitochondrial function.^64^, ^65^ While conventional cardiovascular and heart failure treatments have shown promising results in the treatment of cardiac cachexia patients as well as preclinical cancer‐induced cardiac cachexia, there is still no clinical standard of care nor treatment aimed specially at cardiac wasting and dysfunction in cancer survivors.

### 3.2 Emerging pharmacological targets and treatment strategies for cancer‐induced cardiac cachexia

**FIGURE 5:**
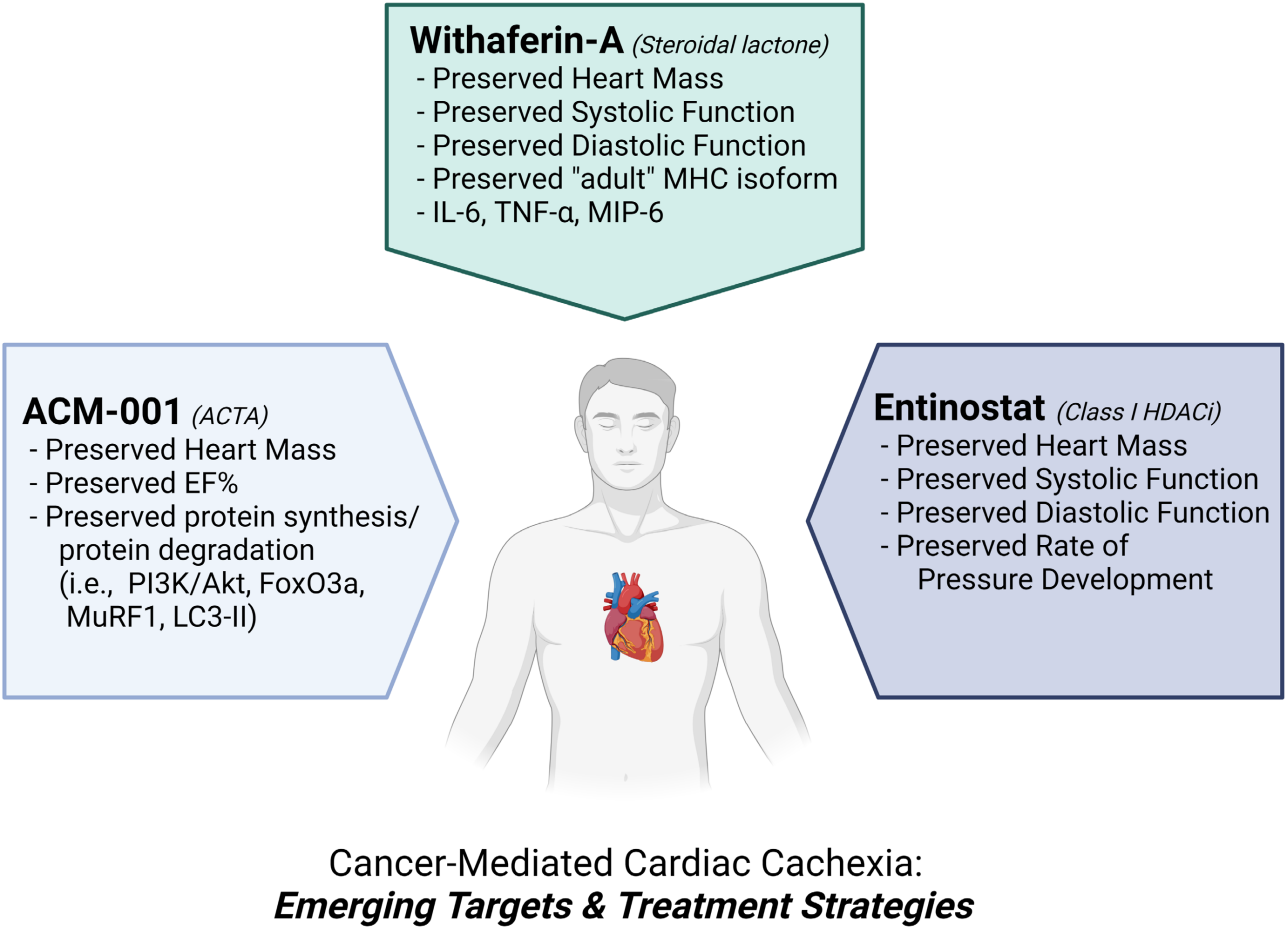
Emerging targets and treatment strategies for cancer‐mediated cardiac cachexia. In recent years, a handful of therapeutic targets and treatment strategies have emerged. ACM‐001 (anabolic‐catabolic transforming agent [ACTA], also known as espindolol), withaferin‐A (steroidal lactone), and Entinostat (Class I histone deacetylase inhibitor [HDACi]) have shown success in treating cancer‐induced cardiac cachexia in preclinical models as well as early clinical trials.

Lesser known supplements and pharmacological treatments, such as withaferin A, ACM‐001, and histone deacetylase inhibitors, have shown promising results as emerging treatment strategies against cancer‐induced cardiac cachexia in preclinical models (Figure 5). Withaferin A, known as a prehistoric remedy in Ayurveda, has been found to have anti‐inflammatory and anticancer healing potentials.^66^ Recently, withaferin A has also shown treatment potential for cancer‐induced cardiac cachexia in a preclinical ovarian cancer‐induced cachexia model.^41^ The pharmacological treatment resulted in significant preservation of cardiac weight, systolic function, and diastolic function. Additionally, withaferin A treatment in tumor‐bearing mice also led to significantly decreased levels of pro‐inflammatory cytokines TNFα, IL‐6, and MIP‐6 in the heart, and it prevented the metabolic MHC shift of cardiomyocytes from an “adult” to “embryonic” isoform that was seen in tumor‐bearing animals.^41^ Similar findings were seen in a mouse model of breast cancer‐induced cachexia and endothelin receptor blocker treatment. Tumor‐bearing mice that were treated with atrasentan showed significant improvements and preservation of LV structure and function that was not achieved with commonly used beta‐blockers.^67^

ACM‐001, a new anabolic–catabolic transforming agent (ACTA), has previously been shown to have positive effects on cancer cachexia by reversing sarcopenia and muscle wasting as well as improve grip strength and quality of life.^68^ In fact, ACM‐001 (espindolol) was tested in a phase II clinical trial in cancer cachexia patients and showed success in maintaining fat mass and muscle strength.^69^ Pötsch et al.^68^ further investigated ACM‐001 role in cancer cachexia compared to commonly used beta‐blockers, carvedilol, metroprolol, nebivolol, tertatolol. Male Wistar Han rats treated with ACM‐001 and inoculated with Yoshida hepatoma AH‐130 cells showed significant reductions in skeletal muscle wasting, prevention of LV mass wasting, as well as improved ejection fraction and fractional shortening compared to non‐treatment tumor‐bearing rats and rats treated with beta‐blockers. In fact, rats treated with high doses of tertatolol and low doses of carvedilol experienced significantly increased risk of mortality, indicating that beta blockers may not be the most beneficial treatment strategy for cancer‐induced cardiac cachexia. Additionally, ACM‐001 treatment seemed to attenuate homeostatic imbalances of protein synthesis and degradation (i.e., PI3K/Akt, FoxO3a, MuRF1, LC3‐I/II) in the hearts of tumor‐bearing animals.^70^

Certain types of a relatively new class of anti‐cancer treatment, histone deacetylase inhibitors (HDACi), have also shown promising results on attenuating cardiac complications in B16F1 and LLC tumor‐bearing mice. Histone deacetylase inhibitors have recently been approved by the FDA for use in cancer patients, but the underlying mechanisms remain understudied.^71^ Bora et al.^72^ have found that Class I HDACi Entinostat treatment was successful in preserving cardiac muscle mass, structure, and function (systolic pressure, diastolic pressure, rate of pressure development), therefore attenuating cardiac complications associated with cancer cachexia in both B16F1 and LLC tumor models.^72^ These findings suggest that further research is critically needed to understand the underlying mechanisms of these pharmacological treatments as well as their effects on cancer‐induced cardiac cachexia.

### 3.3 Non‐pharmacological treatment of cancer‐mediated cardiac cachexia

Targeting improvement of appetite via medication, such as megestrol acetate, or via nutritional interventions is another approach that has shown benefits in the treatment of cancer‐induced cardiac cachexia. Megestrol acetate administration in Yoshida AH‐130 rats has demonstrated significant improvements in ejection fraction and fractional shortening by modulating autophagic pathways.^3^, ^73^ It has also been suggested that implementation of individualized, non‐pharmacological, nutritional interventions may be beneficial in preventing or slowing the development of this disease. Preclinical studies on cachectic mice have shown that a dietary intervention with lauric acid and glucose might improve cardiac dysfunction and remodeling due to cancer‐mediated cardiac cachexia.^74^

Another possible cost‐effective and accessible non‐pharmacological treatment option is exercise in the form of aerobic endurance exercise or resistance exercise (Figure 4). Due to the anti‐inflammatory effect, specifically aerobic endurance exercise can act as an anti‐inflammatory promoter by inducing the release of anti‐inflammatory cytokines, such as IL‐10. The release of IL‐10 can reduce systemic inflammation, decrease protein degradation, and increase protein synthesis, while also regulating insulin signaling and metabolic remodeling of the cardiac tissue.^2^, ^19^ The management of muscle wasting and maintenance of muscle mass during cancer cachexia are two factors that significantly increase prognosis of survival.^75^ Therefore, exercise in any mode or intensity might have the ability to preserve muscle mass by attenuating atrophy via modulation of metabolic and inflammatory pathways. This can ultimately aid in improving tolerance to physical activity, decreasing fatiguability, enhancing lifespan, and increasing quality of life.^76^ Several preclinical studies have indicated that exercise, in the form of short‐term high intensity exercise, moderate intensity treadmill exercise, or wheel running, can partially reverse LVEF, reduce cardiac remodeling, and increase LV mass, while also modulating the expression of autophagic markers resulting in decreased cardiac necrosis, fibrosis and inflammation.^36^, ^42^, ^77^ While there is not as much data on the benefits of resistance training, it has been shown that resistance exercise might stimulate the activation of mTOR signaling to induce protein synthesis. This mechanism could regulate the imbalance of protein degradation and synthesis and preserve muscle strength and mass.^78^ The most effective timing, duration or intensity of exercise has not yet been identified and the isolated use of physical exercise may not be sufficient in preventing or reversing muscle wasting.^7^

While there are promising non‐pharmacological and pharmacological treatment options that could potentially be used to overcome the detrimental effects of cancer‐induced cardiac cachexia, the most effective combination of treatments has not been established and further research needs to be done to develop individualized treatment plans including multimodal approaches of pharmacological, dietary, and exercise interventions.

## 4 CONCLUSION

Most cancer patients suffer from a complex metabolic wasting disease known as cancer cachexia, especially at advanced stages of certain cancers. Muscle wasting, cardiac remodeling, and cardiac dysfunction are the main symptoms that patients with cancer‐induced cardiac cachexia suffer from, ultimately leading to heart failure and death in most patients. Cardiac atrophy and dysfunction results from an abnormally altered metabolism (e.g., MHC shift, insulin deficiency), leading to an imbalance in protein synthesis and degradation, increased systemic inflammation via upregulation of IL‐6 and TNFα, as well as altered energy availability. The underlying metabolic mechanisms are not fully understood and there are no clear diagnostic criteria or effective treatment options to reverse or attenuate the detrimental effects of cancer‐induced cardiac cachexia. While researchers have identified potential biomarkers to detect or monitor the progression of the disease, further research is needed to be able to identify the development and stage of cardiac cachexia successfully and reliably in cancer patients. The combination of pharmacological and non‐pharmacological therapies, in the form of dietary and exercise interventions, has shown the most effective results in preclinical and clinical trials. However, cancer stage, lifestyle, physical fitness, and psychological factors need to be considered when implementing therapy options, suggesting that an individualized treatment plan for each cancer patient may be most effective to prevent, inhibit, or potentially reverse the detrimental effects of cardiac and cancer cachexia.

## AUTHOR CONTRIBUTIONS

**Louisa Tichy:** Conceptualization (equal); data curation (equal); formal analysis (equal); visualization (equal); writing – original draft (equal); writing – review and editing (equal). **Traci L. Parry:** Conceptualization (equal); data curation (equal); formal analysis (equal); visualization (equal); writing – original draft (equal); writing – review and editing (equal).

## FUNDING INFORMATION

Traci L. Parry is funded by University of North Carolina Greensboro.

## CONFLICT OF INTEREST STATEMENT

None of the authors have any conflict of interest to disclose.

## Data Availability

Data sharing is not applicable to this article as no new data were created or analyzed in this study.
